# A Sustainable Path for Automotive Composite Tooling: Novel Materials, Design, and Technologies Through FEM and LCA

**DOI:** 10.3390/polym18060757

**Published:** 2026-03-20

**Authors:** Gloria Anna Carallo, Laura Magnasco, Stefano Chiocca, Andrea Lessio, Michela Mattia, Michele Morbarigazzi, Alessio Verdulli

**Affiliations:** 1 ESG, Sustainability, and Climate Risk, RINA Consulting S.p.A., 16129 Genoa, Italy; laura.magnasco@rina.org; 2 Aerospace & Defense R&D, RINA Consulting S.p.A., 16129 Genoa, Italy; stefano.chiocca@rina.org; 3 Mechanical Design and Simulation, RINA Consulting S.p.A., 16129 Genoa, Italy; andrea.lessio@rina.org; 4 Sustainability, Decarb and Materials R&D, RINA Consulting S.p.A., 16129 Genoa, Italy; michela.mattia@rina.org; 5 R&D Management, Bercella S.p.A., 43040 Varano de’ Melegari, Italy; m.morbarigazzi@bercella.it; 6 Automotive & Motorsport, Bercella S.p.A., 43040 Varano de’ Melegari, Italy; a.verdulli@bercella.it

**Keywords:** carbon-fibre composites, sustainable materials, additive manufacturing, life cycle assessment, finite element analysis, polymer recycling, automotive

## Abstract

In the automotive industry, the push for lightweighting, sustainability, and performance underpins the need for continuous improvement of materials and processes; thus, this research explores the introduction of different approaches for processing optimization. The Finite Element Method (FEM) excels at enhancing structural efficiency and reducing material use in composite tooling like stamping dies, while Life Cycle Assessment (LCA) quantifies environmental impacts over the product life cycle. Coupling these approaches is promising but challenging due to difficult integration into well-established industrial practices. In this framework, the study presents the combination of FEM-LCA analyses on a tool for a composite car bonnet, considering an industrial case. The reduction in weight (−85%) obtained through FEM topology optimization, along with novel materials (thermoplastic polymers) and processes (3D printing, internal recycling), results in an environmental impact reduction over the tooling process (−43% in climate change). The two analyses enable a holistic tool design that balances mechanical performance with reduced carbon footprint, aligning with the European regulatory framework and emission targets. The results demonstrate the feasibility of a coupled FEM-LCA approach to optimize composite tooling in the automotive context, with a positive prospect of full-scale integration into the industrial value chain.

## 1. Introduction

Fiber-reinforced polymer (FRP) composites, like carbon fiber- (CFRP) and glass fiber-reinforced plastics (GFRPs), are increasingly deployed in automotive structures to meet stringent demands for lightweighting while preserving safety and stiffness [1]. Indeed, their high strength-to-weight ratios, corrosion resistance, and design flexibility make them appealing alternatives to metals [2], as seen in vehicles from different market segments, from BMW to Lamborghini [3]. In addition, stricter CO_2_ regulations—including the European Union’s 95 g/km target [4]—drive innovations in automotive composites. However, metal-to-composite transitions introduce challenges, including resource-intensive production of carbon fibers and thermosets, which can undermine their advantages [5].

Industrial processes have shifted from labor-intensive hand lay-up to higher-throughput techniques like resin transfer molding (RTM), compression molding, and automated fiber placement (AFP), but premium CFRP parts (with custom, low-batch runs) often rely on autoclave bag molding due to scalability and cost issues [5]. Moreover, recycling still remains critical for epoxies, with most thermoset-based CFRPs ending up in landfills [6] and posing additional sustainability concerns, especially given energy-intensive autoclave curing and volatile organic compound (VOC) emissions during lay-up [7].

In this context, accurate prediction and control of the thermo-mechanical behavior of composite tools are crucial to guarantee dimensional tolerances, reduce scrap, and shorten cycles. Finite Element Method (FEM) models became the de facto approach to simulate the thermo-mechanical response of the mold during the curing cycle and service life. Indeed, recent studies demonstrate the suitability of FEM-based simulations for evaluating temperature gradients, resin conversion, and residual stresses in automotive parts [8]. At the same time, researchers combined FEM simulations with topology optimization and machine-learning tools to explore non-intuitive internal geometries and infill patterns while satisfying autoclave constraints [9].

Besides material optimization, increasing concerns about the environment led to the introduction of sustainability studies early at the design stage. Life Cycle Assessment (LCA) quantifies environmental impacts by compiling mass and energy flows over raw material production, tooling fabrication, curing, and end-of-life management, revealing composites’ intrinsic footprint. For automotive CFRP components, cradle-to-gate LCA observes that production phases typically dominate impacts (60–80% of Global Warming Potential) [10], with precursors (e.g., polyacrylonitrile (PAN) processing for carbon fiber) and process energy being the main contributors [11]. In the last stage, recycling routes and matrix system selection critically affect end-of-life burdens: indeed, the use of thermoplastic CFRP could cut end-of-life impacts by 30% [12]. LCA studies follow ISO 14040/44 standards [13,14], which provide methodologies for sustainability analyses; in automotive contexts, LCAs often inform material selection considering energy consumption and long-term durability [15].

Recent frameworks began to integrate FEM-based performance assessment with LCA-driven environmental screening at the design stage, supporting multi-criteria selection of materials, geometries, and processes for lightweight automotive components [16]. For instance, a combined FEM-LCA approach proved to effectively reduce composite part dimension and process impacts without compromising component quality [17], but research is still at the academic level and the literature on the topic is sparse; consequently, the combined integration of FEM and LCA is rather unexplored in the current industrial practice.

In this framework, the Tool4Life project [18], launched in 2022 by four industrial and R&D partners, received funding by the European Commission through its Life 2021–2027 program. The project tackled sustainability in composite manufacturing by developing thermoplastic recyclable tools for automotive composite parts using additive manufacturing (AM) molds. A multi-disciplinary approach—combining automotive part fabrication, materials research, and processing innovation—was assisted by modeling and sustainability studies to support the innovative tooling design. This paper presents one of the outcomes of the project, by coupling FEM and LCA studies in composite tooling for the automotive sector.

The paper is structured into five sections: Section 1 provides background context for FEM and LCA studies in the field, Section 2 explores tooling materials and processes and FEM-LCA methodologies, Section 3 presents a case study in the automotive sector, Section 4 discusses the topological design and environmental results for the selected tool, and, finally, major remarks and future perspectives are drawn in Section 5.

## 2. Materials and Methods

Firstly, this section describes the traditional composite tooling at BERCELLA (Varano de’ Melegari, Italy), as the technology owner of the products under analysis. The innovative tooling process is summarized, introducing new materials selected by CENTEXBEL (Gent, Belgium) and water-based coatings developed by MARBO (Pogliano Milanese, Italy), both partners of the project. Moreover, the designed recycling process applied to the novel production route is explored.

Secondly, the Finite Element Method (FEM) study is introduced, outlining its intent and structure. Thirdly, the Life Cycle Assessment (LCA) methodology is presented and detailed in its four iterative steps. These methodologies are applied by RINA (Genoa, Italy) on the object of the study, i.e., a tool for a composite car bonnet, in its two configurations (standard vs. innovative).

### 2.1. Production Process for Composite Tooling

Composite parts are traditionally manufactured using molds, commonly referred to as *tools*. Tooling consists of three-dimensional forms that define the final shape of the component. This section details the conventional tooling process, involving master models and traditional machining.

#### 2.1.1. Conventional Tooling Process

**Figure 1:**
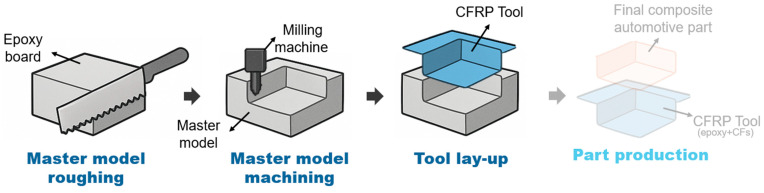
Conventional tooling (Business-as-Usual, BAU scenario)—process scheme.

The common practice in composite tooling relies on subtractive manufacturing and requires a preparatory step with master models (also called *plugs*). These forms are typically produced from large blocks of foamed materials, most often thermoset epoxies or polyurethane foams with high density. The shaped block (*master model*) is then used to produce composite tools. Overall, this approach represents a well-established solution for tooling in the composite industry, especially for top-level composite components, with low-batch and customized runs, where production is driven by adaptability to accommodate customer-specific demands. In Figure 1, a schematic of the conventional tooling process is outlined; the final composite part production is outside the scope of this study.

**Figure 2:**
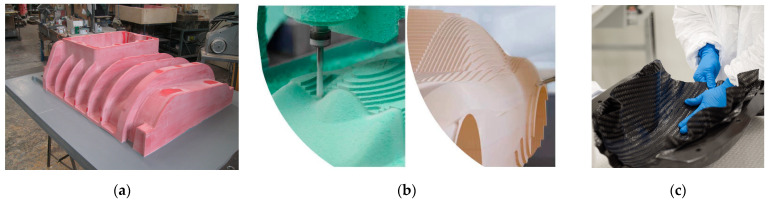
Conventional tooling process (BAU) steps: (**a**) master model—foam blocking; (**b**) master model—CNC machining; (**c**) composite tool—hand lay-up (courtesy: Bercella).

Conventional tooling begins with the preparation of a master model in thermoset resin. Firstly, 3D Computer-Aided Design (CAD) modeling captures the component’s geometry, draft angles, and machining allowances. Then, the parameters driving the bulk material selection are density, strength, stability, and machinability. The foam blocking strategy is planned, determining the slab arrangement and volume assembly strategy. Next, foam blocking cuts and bonds foam slabs with structural adhesives, which are then cured to form a solid homogeneous block (Figure 2a). Once ready, the block is shaped using Computer Numerical Control (CNC) roughing to remove excess bulk material, and CNC machining to create the final inner shape for tool lay-up, matching the CAD design (Figure 2b). Finally, in surface preparation, a sealer is used to fill intrinsic foam porosity and create a smooth and resistant outer layer, while a release agent is applied onto the master model to allow an easier separation of the tool in the second stage.

Conventional master model production has evident limitations: thermoset polymers are commercially available only as standard blocks; hence, the fabrication of large or complex tools leads to significant material waste from the oversized blocks. Extended procurement times are also encountered because pre-customization of epoxy blocks is uncommon and expensive, preventing the application of FEM-based geometry optimization on baseline tooling.

Once the master model is finalized, a conventional tool is made via hand lay-up using low-temperature curing epoxy prepregs, in order to have a match with the master model on a technology parameter, i.e., the coefficient of thermal expansion (CTE). Hand lay-up stacks multiple prepreg plies in a sequence over the master model shape (Figure 2c): an initial lightweight fabric ply (160–200 g/m^2^) ensures surface fidelity; then, symmetrical structural plies (0°/90°, ±45°) minimize stresses and warpage. Then, in *vacuum consolidation*, the prepreg stack is put under a vacuum bag (pressure = −0.9 bar), with a pressure decay (<5 mbar/min) to prevent porosity. The *curing* process in the autoclave usually undergoes a curing cycle with the following parameters:

- Heating ramp at 20 °C/hour until reaching 180 °C (approximately 8 h);
- Dwelling at 180 °C for 4 h;
- Cooling down to 60 °C at a controlled rate between 1 °C and 5 °C/min.

In the end, the CFRP cured tool is gradually cooled down (<40 °C) to mitigate CTE-induced stresses between the tool’s carbon fiber and the master model materials. The tool is eventually machined, and its surface is prepared (e.g., gelcoat polishing, release agent) to ease final part de-molding.

Globally, the conventional tooling method provides a reasonable cost-to-size ratio and good geometric accuracy, but it exhibits notable drawbacks such as longer procurement and production lead times (several days, because of multiple sequential stages), high material loss in production (e.g., due to the oversized starting blocks), non-recyclability of thermoset materials, and thermal sensitivity variation with foam type. An alternative solution to baseline tooling involves direct printing, as proposed in the Tool4Life project.

#### 2.1.2. Innovative Tooling Process

**Figure 3:**
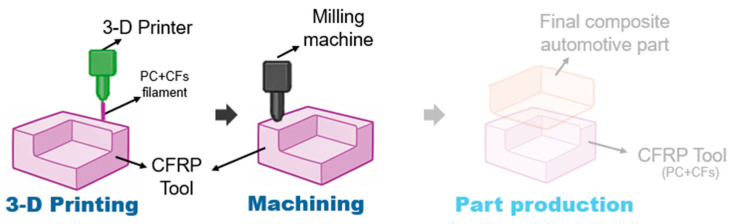
Innovative tooling (Tool4Life, T4L scenario)—process scheme.

The innovative tooling process relies on the use of a large-scale 3D printer known commercially as kreator ares™ by CMS (Zogno, Italy). This tool works with a 3D extrusion-based process, which enables the creation of large-dimension and complex-shape components in a single step, reducing production time and minimizing the mass of the printed piece through prior FEM optimization. Besides the simplification of the tooling process and timing—thanks to the absence of the master model, faster tooling through near-net shape 3D printing, and very minor mill refining—the selection of more sustainable raw materials is possible: indeed, thermoplastic filaments (e.g., polycarbonate (PC), acrylonitrile butadiene styrene (ABS)) with CF loading are used to obtain recyclable CFRP tools. In Figure 3, a schematic of the innovative tooling process is illustrated: again, the final component production is not part of this study.

**Figure 4:**
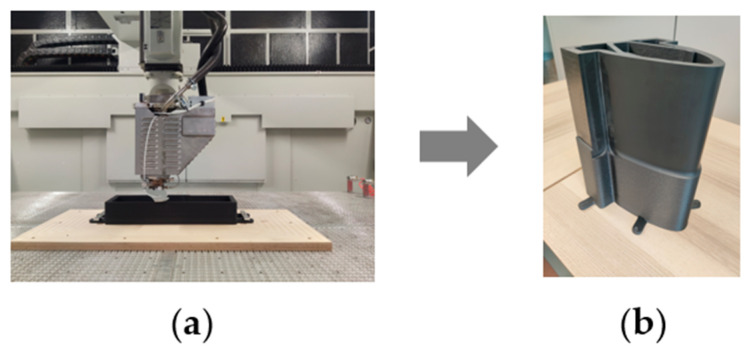
Innovative tooling process (T4L): (**a**) tool 3D printing; (**b**) composite 3D-printed tool (courtesy: Bercella).

The Tool4Life (T4L) tooling process begins with the FEM optimization of initial tool geometry (mass reduction as a driving parameter), which is then fed into a specific slicing software (ICARUS™ sourced by CMS) required for the model 3D printing. Here, printing parameters (e.g., nozzle temperature, printing speed) are tailored with thermoplastic material properties (e.g., viscosity, melting temperature, etc.) to achieve the desired ultimate characteristics of the tool. Once the parameters are set, the 3D printing process is launched (Figure 4a); for processing such large tools or parts, a printing time of 3 to 14 h is required, in a specific climate chamber. Thanks to the 3D printing process, unnecessary volumes can be left hollow, reducing weight and offering a distinct advantage over traditional milling of solid blocks. In terms of starting materials, a thermoplastic pellet batch can be used for every tool, without size/geometry constraints and lead time procurement issues.

At the end of the printing process, the near-net shape tool is detached from supports (wood board), and milling is performed to remove the excess material designed to meet the dimensional specifications of tolerance standards (Figure 4b).

At the end of their life cycle, composite tools must be stored and disposed of, presenting the additional challenge of waste management. While for thermoset materials (BAU scenario), their final fate is often landfill, producing them from thermoplastics enables material recovery and recycling, including their reusability for 3D printing (internal recycling). Indeed, thermoplastic recycling is integrated into the innovative tooling process. At their end of life, tool parts are shredded to obtain material of suitable size for remelting and are converted into pellets, i.e., the form in which they feed the 3D printer’s extrusion system. The shreds from tool milling are also recycled in the same way. The shredding and re-pelletizing can be performed both on site (with dedicated equipment installed directly beside the printer) or outsourced. Real-time evaluation of material degradation during recycling strongly affects its reusability in real industrial practice.

In conclusion, 3D printing yields lighter tools than conventional multi-step processes, slashing time and material use, thanks to FEM optimization and the absence of a master model. Overall, the T4L method is characterized by greater flexibility; moreover, the use of thermoplastics enables recovery, recycling, and reuse of tools.

### 2.2. Finite Element Method (FEM)

The Finite Element Method (FEM) is central to topology optimization, discretizing the design domain into finite elements to simulate structural responses like stress and displacement under given loads and constraints. Hereafter, the methodology and its application to the study are described.

#### 2.2.1. FEM Methodology

**Figure 5:**
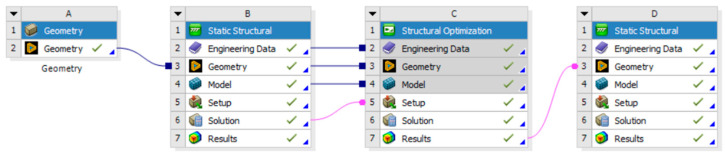
Procedure for topological optimization (courtesy: Ansys).

Tool geometry optimization is the first step of the integrated FEM-LCA approach discussed in this study. A common procedure for the topological optimization of the chosen geometry is carried out on dedicated FEM software, as outlined in Figure 5, and it is divided into several steps: (1) the initial geometry (A) of the tool is imported and, if necessary, it is simplified or cleaned from elements that are not useful for optimization and structural simulation purposes; (2) in this sub-step (B), the mechanical characteristics of the material, the mesh, and the boundary conditions (constraints and loads) are introduced and the analysis is launched; (3) in loop with the previous step, the phase (C) allows for the removal of material that contributes less to the mechanical strength of the component; (4) the optimized geometry is then analyzed again in step (D) to verify that the result meets the required specifications.

This general procedure is customized for the specific object of the design process, i.e., the tool for a composite car bonnet, as described in the following section.

#### 2.2.2. FEM Applied to the Study

**Figure 6:**
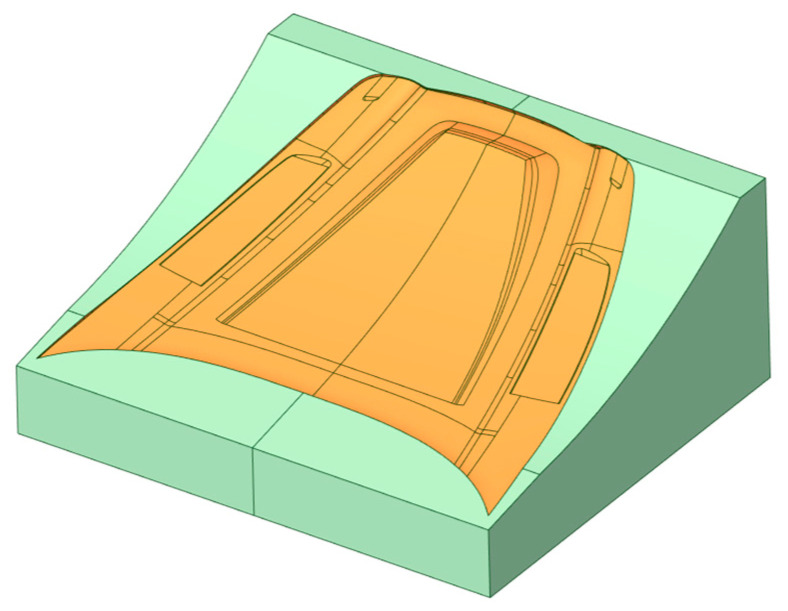
Initial tool geometry.

This paragraph presents the characteristics of the FEM simulation related to the tool for an automotive part (i.e., car bonnet in composite material). The optimization is carried out using well-known FEM software, i.e., Ansys Workbench© (v. 2024 R2), sourced by Ansys™ (Canonsburg, PA, USA) [19]. Figure 6 shows the initial tool geometry, where the functional surface of the tool (i.e., the engraved zone where the final car bonnet will be hand-laid-up) is highlighted in orange, while the remaining part of the tool is in green.

After the geometry definition, the material properties are defined in the software library. In particular, a thermoplastic polymer matrix, polycarbonate (PC), reinforced with 20% carbon fiber (CF) is selected; the properties used in the modeling, derived from the technical datasheets of the modified PC with CF addition, are reported in the following Table 1.

Finally, the boundary conditions of the simulation are defined. In particular, the production process of composite material objects includes a stage in an autoclave, a specific environment that enables the curing of the resins by impregnating the carbon fiber. However, the effects of pressure and temperature also act on the tool, which is therefore exposed to a harsh environment. For this reason, in the simulation, a pressure of 6 bar (600 kPa) and a temperature of 120 °C are applied to the tool. In addition, gravitational acceleration (g = 9.81 m/s^2^) and a system of constraints are applied to simulate the support on the autoclave base without restricting the part in other directions, allowing it to move freely due to thermal expansion.

The optimization process aims to reduce the amount of material used to manufacture the mold, while still meeting the maximum allowable deformation criteria, which are verified in the final phase (phase D). For this reason, only mechanical variables are considered during the optimization process.

The structural optimization phase acts on the entire internal volume, excluding the face where the ‘functional’ part of the mold is located, as well as the vertical front and rear faces (Z direction). The optimization follows a density-based approach and uses compliance minimization as the single-parameter objective function. The optimization constraints include a retained mass below 30% and a manufacturing constraint imposing a minimum wall thickness of 50 mm. By reiterating the optimization calculation with an increased mass constraint, the configuration with the lowest mass still meeting the required characteristics (maximum deflection) is identified.

The optimized results are then verified in the (D) module, in which the new geometry is again subjected to all the boundaries described for the (B) module.

### 2.3. Life Cycle Assessment (LCA)

Environmental studies like LCA have gained recognition over the past few decades, proving their success and applicability in several research and industrial sectors, both as ex ante (e.g., eco-design) and ex post (i.e., product footprint) assessment studies. Below, a description of the methodology and the application to the present study is reported.

#### 2.3.1. LCA Methodology

Life Cycle Assessment (LCA) is a comprehensive and internationally standardized methodology aiming at the evaluation of the environmental impacts associated with products, processes, or services over their life cycle [12]. This method identifies and quantifies materials and energy consumptions, process emissions in the environment, and waste disposal related to the life cycle (as a whole or as a part of it) of the object of the study.

LCA is ruled by a set of international standards, e.g., ISO 14040:2006 [13] and ISO 14044:2006 [14], and also guidelines (e.g., ILCD Handbook [20]), which illustrate the procedures, intent, and structured steps to conduct the assessment. According to these norms, LCA is articulated in four iterative phases: (1) goal and scope definition, which sets the premises and parameters of the analysis, including the object of the study (i.e., a product, a process, a service), the functional unit (FU), i.e., the metric (i.e., parameter, value, unit), which acts as a reference for data and results, and the system boundaries within which the study is carried out; (2) Life Cycle Inventory (LCI), listing process data including both inputs (e.g., energy, raw materials) and outputs (e.g., product, waste, emissions), as well as background (secondary, generic) and foreground (primary, specific) process data; (3) Life Cycle Impact Assessment (LCIA), where LCA software (e.g., GaBi^®^, SimaPro^®^) reproduces the model and calculates the environmental impacts as a set of indicators, each for a specific environmental aspect (e.g., ecotoxicity, ecosystems, resource use) [21]; and (4) interpretation of results, where the main hotspots are identified and validated, enabling us to draw major remarks and lessons learned from the study.

This general and standardized approach is customized for the specific topics and framework of the present study, as discussed in the next section.

#### 2.3.2. LCA Applied to the Study

In this study, the goal of the LCA is to compare the conventional and the innovative tooling processes after FEM optimization. Hence, the scope is to conduct a comparative assessment between two scenarios for the same product (i.e., the tool) under analysis. In the conventional tooling process (Business-as-Usual, BAU scenario), an epoxy master model is first roughed and machined; then, the composite tool itself is hand-laid-up on it. In contrast, the innovative process (Tool4Life, T4L scenario) directly shapes the optimized tool with 3D printing, without any need for a master model.

The comparative LCA study adopts the following settings:

- Object of the study: a tool for a composite part production;
- Functional unit (FU): an ***n.1 tool for an automotive composite part (*i.e., *car bonnet)***;
- System boundaries: from production until disposal or recycling of the tool (i.e., cradle-to-grave approach); five cycles of tool production evaluated in both scenarios;
- Exclusions: the use phase of the tool is the same in both scenarios (same functionality); similarly, composite part (car bonnet) production is excluded, since it is not the object of innovation or modification between the scenarios;
- Allocation of master model impacts: as the master model is reused five times in the traditional scenario, one-fifth of its production and disposal impacts are assigned to the tool’s life cycle;
- Allocation of recycling impacts: 50:50 allocation of recycling impacts between material end of life and recycled material input in the innovative scenario;
- LCA software: LCA for Experts (Gabi^®^) v.10.9 [22], sourced from Sphera™ (Chicago, IL, USA), including the ecoinvent© (Zurich, Switzerland) v3.10 database [23];
- Impact assessment: Environmental Footprint (EF) 3.1 Life Cycle Impact Assessment method [24].

## 3. Case Study: Composite Tool for a Car Bonnet

**Figure 7:**
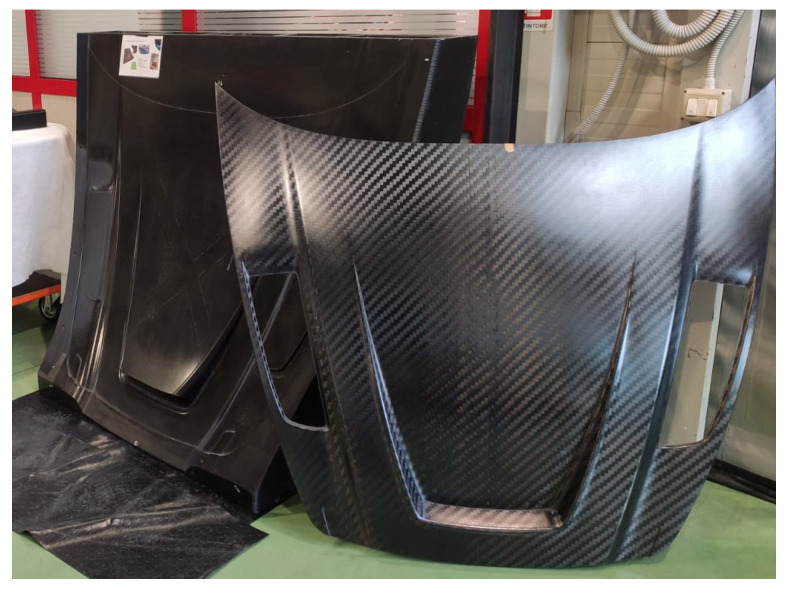
Case study—tool for a car bonnet (back: tool; front: final car bonnet) (courtesy: Bercella).

The case study selected to validate the innovative tooling method is a tool for producing a car bonnet (Figure 7). This geometry is chosen because of its particular dimensional, aesthetic, and aerodynamic requirements. The study focuses on comparing the advantages of creating the same tool via traditional technology—epoxy master model blocks on which the CFRP tool is hand-laid-up—against an innovative tooling process through 3D printing after FEM topology optimization of the item.

From a technological point of view, differences between the approaches are substantial, where crucial optimization parameters are mass input, waste output, and process time. Traditional tooling uses an epoxy board with a density of 0.69 g/cm^3^ for the master model and an epoxy-based CFRP prepreg of 1.52 g/cm^3^ for the tool, while the innovative process employs only a polycarbonate CF-loaded filament with a density of 1.27 g/cm^3^. The total raw material input is strongly decreased in T4L processing thanks to the absence of a master model: despite the tool being slightly heavier in the T4L scenario (also due to material change), a global material input saving of −66% is achieved. Moreover, total scrap waste sees a dramatic drop from 721 kg (including the master model) to 30 kg (for the 3D-printed item), i.e., a 96% waste reduction benefiting disposal costs and ecological impact. Milled scraps are limited thanks to geometry optimization; in addition, the thermoplastic shreds are sent to recycling. The mechanical performance and recyclability of thermoplastics are experimentally investigated with repeated melting, re-processing, and re-printing up to five times, confirming that the polymer matrix retains its capacity to be melted and reprocessed repeatedly.

Processing time is reduced from 60 h to 20 h—a 67% decrease, with significant energy savings (e.g., no autoclave curing). The specific 3D printing parameters adopted in this study are not reported in detail, as they constitute confidential know-how of the customized industrial process. The machinability of the thermoplastic material through CNC milling was also directly validated during project trials, obtaining high-quality tooling surfaces and good compatibility with the innovative water-based sealer. Results on effective surface finishing and part de-molding confirmed the full applicability of the thermoplastic-based CFRP tool under industrial conditions.

The service life of tooling is defined in terms of potential reusability rather than in a timeframe. Both BAU and T4L tools are designed to provide the same functionality, i.e., enabling the manufacture of a composite car bonnet; hence, the equivalence is validated with project demonstrators and allows for a consistent comparison in this study.

From an FEM design perspective, the tool shape is optimized (an 839 kg full block to a 126 kg final shell) while maintaining the same strength. As trials demonstrated, the use of a thermoplastic material instead of an epoxy-based CFRP does not alter the desired performance of the tool, which was the final goal of FEM optimization. From an LCA point of view, the functionality and use of the tool are kept constant (hence, the use phase is neglected) in both scenarios for a fair comparison. The master model (BAU) is assumed to be reused five times before disposal, so its burdens are appropriately scaled, with a more conservative and realistic approach (as the impacts of the BAU scenario per tool are lowered). Major differences concern materials and processes: the thermoset BAU master model and tool are both sent to the landfill, while the thermoplastic T4L tool (including shreds) can be internally recycled.

Table 2 summarizes the materials and characteristics of tooling processes between the compared scenarios.

## 4. Results and Discussion

This section details the results of the FEM-LCA integrated approach on the object of the study, i.e., a tool for a composite car bonnet. The optimization achieved through FEM is presented first, and then the LCA analysis assesses the environmental profile of the T4L optimized tool with respect to its baseline (BAU) configuration.

### 4.1. FEM Optimization

The results of the optimization procedure described in Section 2.2.1 and Section 2.2.2 can be divided into structural results (i.e., stresses and displacements) for steps (B) and (D) of the FEM approach, and material reduction for step (C). The structural analysis, based on finite elements, allows for the replication of the physical conditions present in the autoclave, namely temperature (T = 120 °C) and pressure (p = 600 kPa), as well as a set of displacement constraints that allow the mold to rest on the ground without the need for artificial constraints in the other directions. The topology optimization includes a numerical phase in which the sole parameter of the objective function (structural compliance) enables, through a density-based approach, an iterative calculation of mass reduction. A structurally acceptable configuration is reached (i.e., displacements are kept below the tolerance threshold). In addition to this, a fundamental tooling step for the mold is carried out; hence, the optimized raw geometry is reviewed in order to adapt it to the printing process.

**Figure 8:**
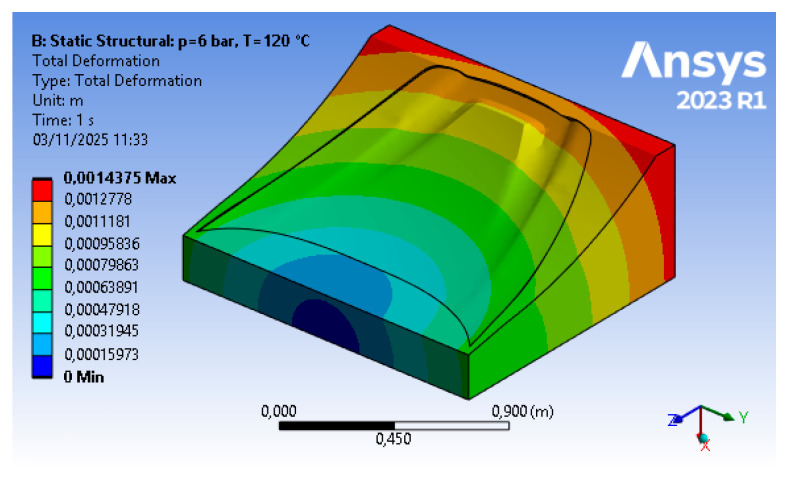
Step 2 FEM results—displacement (courtesy: Ansys).

**Figure 9:**
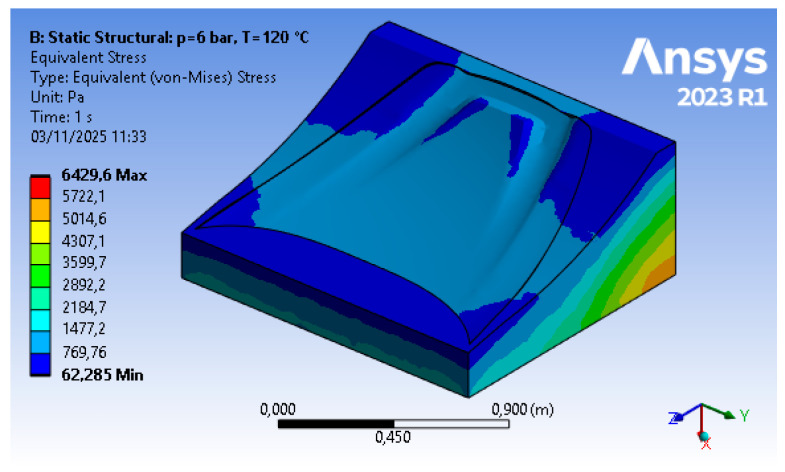
Step 2 FEM results—Von Mises equivalent stress (courtesy: Ansys).

The outcomes of step (B), corresponding to displacements shown in Figure 8 and Von Mises equivalent stress shown in Figure 9, respectively, represent the starting point for the tool optimization. The values reported in these initial results show, under autoclave operating conditions, a maximum displacement of about 1.4 mm and a stress of approximately 6.4 kPa. The object analyzed, in its initial form, exhibits negligible stresses and displacements that lead to deviations within the tolerance limits.

**Figure 10:**
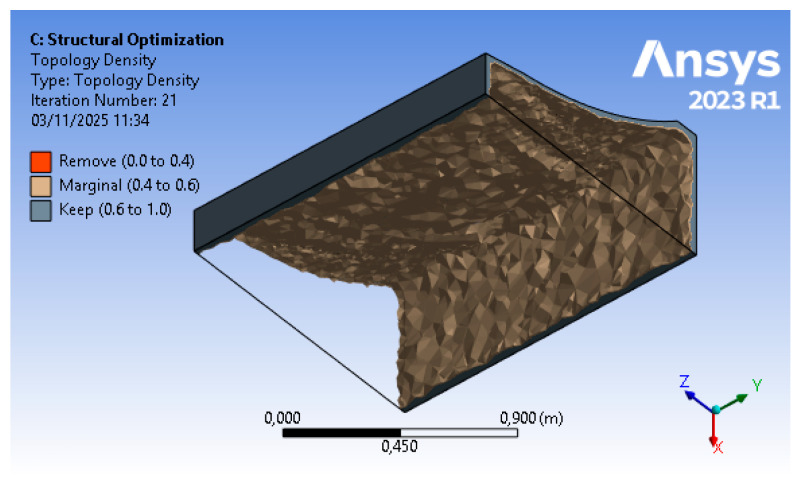
Step 3 FEM results—raw optimized geometry (courtesy: Ansys).

The results of step (C) of the topology optimization include the optimized geometry, shown in its raw form in Figure 10 (i.e., the direct outcome of the numerical process) and later on refined through the CAD process. The initial mass of 839 kg, drawn from the technical specifications of the in-use tool, is reduced to 116 kg in the raw result and to 126 kg in the refined result thanks to FEM topology optimization. The topology optimization process, also considering the low stresses highlighted in the previous point, enables a significant reduction in material usage (up to 85%).

**Figure 11:**
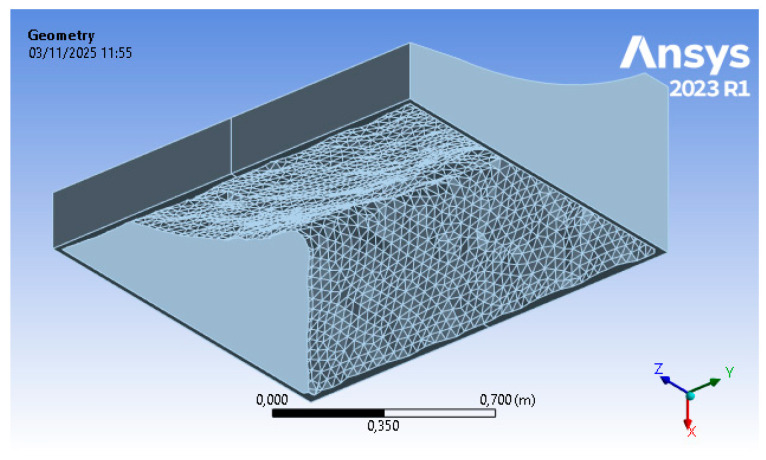
Step 3 FEM results—reviewed optimized geometry (courtesy: Ansys).

The geometry resulting from this optimization (reported in Figure 11) process is therefore validated in the following phase. The step of adapting the geometry from the raw configuration (Figure 10) to the refined one (Figure 11) is a crucial phase in mold development, as it also includes technical aspects that account for practical manufacturing experience. This stage determines the refinement of details that ensure the compatibility of the raw optimized shape with the printing process.

**Figure 12:**
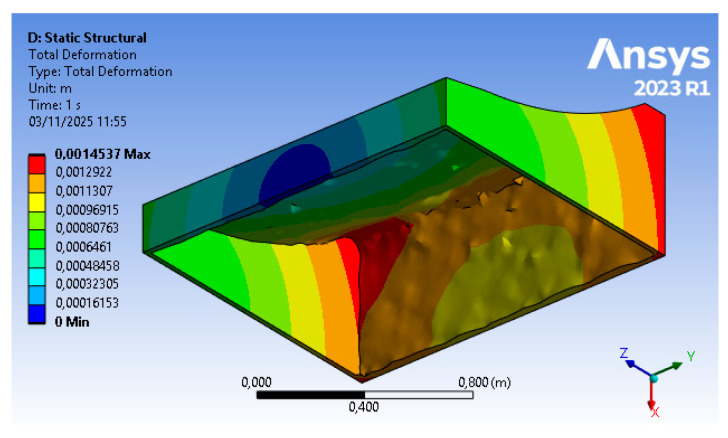
Step 4 FEM results—displacement (courtesy: Ansys).

Starting from the refined geometry, its validation is conducted in step (D), and the results of the finite element structural simulation are reported in Figure 12 for displacement. In this final simulation step, the displacement shows only a minimal change compared to the previous result (from 1.4 mm to 1.45 mm) and therefore remains within the required tolerance limits.

**Figure 13:**
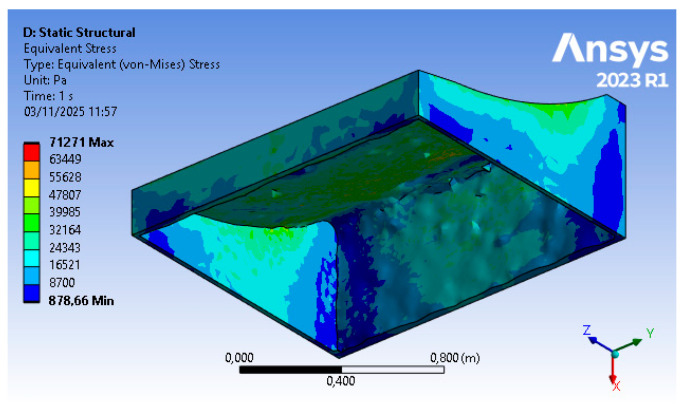
Step 4 FEM results—Von Mises equivalent stress (courtesy: Ansys).

As for the displacements, an increase is also observed for the stresses (71.2 kPa max in areas of numerical concentration), but the maximum distributed value (approximately 40 kPa) remains within acceptable and moderate levels, as outlined in Figure 13.

In conclusion, the final optimized geometry can therefore be considered validated for the intended application of the tool. The numerical procedure enables the design of the mold while maintaining control over its mechanical strength properties and, consequently, over the tool’s deformability. In addition to the purely numerical aspects, challenges were encountered when setting up the mold and in the process of cleaning and adaptation of the raw geometry. This stage involves practical modifications and process-related adjustments.

### 4.2. LCA Analysis

This section presents the results of Life Cycle Impact Assessment (LCIA) of the tool for the composite car bonnet, comparing the baseline (BAU) and innovative (T4L) production scenarios.

Table 3 illustrates the results of the impact assessment for the tool in the baseline BAU and in the innovative T4L configurations. Values are reported for the FU (n.1 tool), and they are expressed for a selection of the most representative and applicable EF 3.1 indicators.

**Figure 14:**
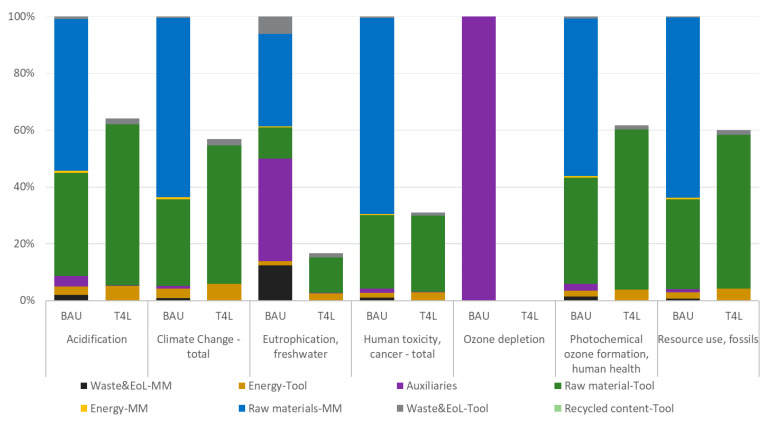
LCA hotspot of a tool for a car bonnet—comparison between BAU and T4L scenarios.

These results are also outlined in Figure 14, where a hotspot analysis is applied to both scenarios, showing the contribution of different inputs and processes.

Going from a traditional to an innovative process results in a significant reduction in all the EF 3.1 selected impact categories. The decrease (i.e., −59% on average) results from the combined application of topology optimization, novel materials, and efficient processes in T4L production. A remarkable improvement is due to water-based auxiliaries, which enable an abatement of *Ozone depletion* category up to five orders of magnitude, whereas their baseline alternatives account for the totality of this indicator because of their organic formulation (e.g., siloxanes and other chemicals). Moreover, the T4L product reports an absolute reduction of −83% and −69% for *Eutrophication*, *freshwater* and *Human toxicity*, *cancer*, respectively, while the emission of CO_2_ eq is reduced from 6 ton to 0.97 ton (−43%) in the *Climate change*, *total* category. Similarly, decreases of −38% and −40% for *Photochemical ozone formation*, *human health* and for *Resource use*, *fossils* reflect the absence of harmful emissions and fossil consumption, respectively.

BAU hotspot analysis demonstrates that the largest share of impacts (>80%) is always due to the raw material value chain; indeed, almost 60% of the impacts are attributable to the master model epoxy board. Moreover, cutting and milling processes are applied to a much larger mass (~1000 kg) than the final master model (~320 kg), with a significant material input to be landfilled. The BAU tool material (i.e., CF epoxy-based prepreg) also has a relative contribution of 31% in Global Warming Potential (GWP, namely the *Climate Change—total* category) and the other categories; finally, recycling content is not possible for BAU thermosets.

In the T4L configuration, most of the impact is due to the thermoplastic filament required for the tool 3D printing. Other T4L life cycle phases are almost negligible because of more sustainable products (e.g., water-based sealant) and processing (e.g., direct shaping, no landfill at end of life). Still, no recycled content is accounted for in this initial T4L cycle, since the chart considers the very first production cycle, with both scenarios having 100% virgin materials as input.

**Figure 15:**
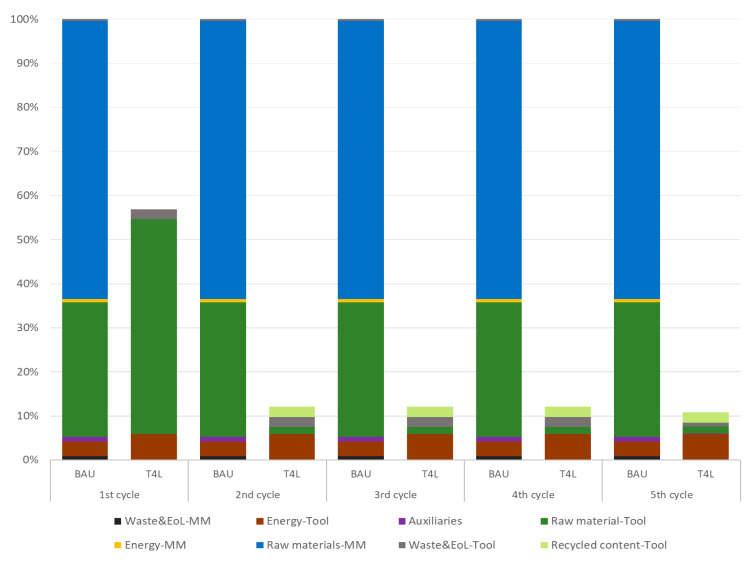
GWP hotspot over 5 life cycles—comparison of BAU vs. T4L car bonnet tool.

The advantage of the innovative T4L production process is further confirmed over time, considering all the five cycles of production, thanks to internal recycling from the second cycle onwards. The decision to use five life cycles results from the research activities carried out during the Tool4Life project, reflecting both the properties of thermoplastic materials (decreasing over the repeated recycling and production cycles, but remaining adequate for the five cycles considered) and the current industrial practices of the company. The results in Figure 15 illustrate a comparison of BAU vs. T4L tool performance over five life cycles for the indicator *Climate Change—total* (GWP).

The first cycle—as already mentioned—reports a −43% for GWP in the T4L product, despite no recycled content being accounted for yet. Going to the second life cycle, the recycled content substitutes 97% of all virgin raw material inputs, hence decreasing T4L impacts up to 86% with respect to the BAU tool. As shown, the baseline scenario is attributed the same impacts in all five life cycles (no internal recycling is possible); hence, for each cycle, BAU tooling is expected to include only virgin materials. On the other hand, in T4L, the same impact distribution is reported for intermediate cycles (from second to fourth), where the production is *closed-loop*; hence, the reduction with respect to the BAU tool is constant (−88% per cycle). The last cycle (fifth), representing the closure of the loop, reports a slight increase in the T4L waste contribution due to the final landfilling of the fifth tool. Overall, a final saving of −89% is the maximum achieved by the T4L tool.

In conclusion, considering the five life cycles, the introduction of the T4L tooling process enables a total GWP reduction of 6.73 tons of CO_2_ eq. per tool, mostly due to the elimination of the need for a master model, improvement in material weight thanks to FEM optimization, high recycled content (reducing virgin raw material input down to 3%), and a significant reduction in landfilled waste.

## 5. Conclusions

The innovations in the automotive industry focus on novel materials and technologies to enhance component mechanical performance while prioritizing sustainability across the life cycle. In this framework, the European Tool4Life (T4L) project developed tooling from thermoplastic materials using additive manufacturing and combined FEM-LCA approaches to successfully enable optimization of tool production and performance. This article discusses the application of an FEM-LCA integrated approach in an industrial case study in the automotive sector.

Initially, FEM analyzed the original tool’s geometry to evaluate structural behavior; then, topology optimization reduced material use while preserving strength. Despite an 85% mass reduction, stresses and deformations stay within tolerance limits for producing composite components, as verified in the final structural checks. Subsequently, LCA compared the FEM-optimized T4L tool against its baseline (BAU) configuration. Key advantages included lower mass from optimization (almost net-shape), elimination of the master model via direct tool 3D printing, thermoplastic materials replacing epoxy CFRP, and recyclability of materials for future cycles. Already in the first production cycle (using 100% virgin material as input), T4L cuts total production burdens, achieving −43% in GWP. With closed-loop internal recycling from the second to fifth cycles, virgin raw material input drops to 3%, boosting savings to −89% in GWP compared to BAU’s linear production.

Finally, results demonstrate that:

- Automotive tools with complex geometry greatly benefit from the mass reduction achieved through FEM optimization in the innovative process;
- Tool mechanical performance is completely retained despite the mass reduction from FEM optimization, maintaining full functionality;
- The improved design, novel materials, and processes are accounted for in the LCA study and are reflected in a contextual improvement of the environmental profile of the tool.

In the end, this study reports a positive and successful application of the coupled FEM-LCA approach in the improvement of design and environmental performance of a case study in the automotive sector. Future developments include the possibility to fully implement this multi-disciplinary approach in industrial practice, transferring the know-how developed with the tool to the final composite parts, in a holistic improvement of the automotive value chain.

## Figures and Tables

**Table 1:** Mechanical properties of the T4L tool material.

Property	Value
Density [kg/m^3^]	1.27 × 10^3^
Coefficient of thermal expansion [1/°C]	1.09 × 10^−5^
Young modulus [Pa]	1.10 × 10^10^
Poisson’s ratio [-]	3.60 × 10^−1^
Tensile ultimate strength [Pa]	1.12 × 10^8^

**Table 2:** Main characteristics of the car bonnet tool in BAU vs. T4L scenarios.

Characteristics	BAU	T4L
Tooling process	master model + tool lay-up	direct 3D printing
FEM optimization	No	Yes
Master model	Yes	No
Master model—material class	epoxy resin foam board	
Master model—material trademark	RAKU-BOARD WB0691	-
Master model—initial mass	1008 kg	-
Master model—final mass	317 kg	-
Tool—material class	prepreg (CFs-Epoxy, 60/40)	thermoplastic CFRP (PC + 20%CF)
Tool—material trademark	CF290 ER450	DAHLTRAM^®^ C-250CF
Tool—final mass	70 kg	126 kg (839 kg before FEM)
Tooling—total time	60 h	20 h
Tool—recycled content	0%	up to 97%
Sealer and release agent	organic-based	water-based
Total waste scraps	721 kg (including MM ^1^)	30 kg
End of life	landfill	internal recycling

^1^ MM = master model.

**Table 3:** LCA results—BAU vs. T4L tool for a car bonnet (FU: 1 tool, cradle to grave).

EF 3.1 Impact Indicators	Unit	BAU	T4L	Variation
Acidification	Mole of H^+^ eq.	2.58 × 10^0^	1.65 × 10^0^	−36%
Climate change—total	kg CO_2_ eq.	1.70 × 10^3^	9.66 × 10^2^	−43%
Eutrophication, freshwater	kg P eq.	2.16 × 10^−2^	3.61 × 10^−3^	−83%
Human toxicity, cancer—total	CTUh	9.23 × 10^−7^	2.86 × 10^−7^	−69%
Ozone depletion	kg CFC-11 eq.	7.23 × 10^−4^	9.65 × 10^−9^	−100%
Photochemical ozone formation, human health	kg NMVOC eq.	2.90 × 10^0^	1.79 × 10^0^	−38%
Resource use, fossils	MJ	3.65 × 10^4^	2.19 × 10^4^	−40%

## Data Availability

The datasets presented in this article are not readily available because of technical limitations due to intellectual property rights (IPRs). Requests to access the datasets should be directed to the authors.

## Abbreviations

The following abbreviations are used in this manuscript:

3D	Tridimensional
ABS	Acrylonitrile Butadiene Styrene
AFP	Automated Fiber Placement
BAU	Business-As-Usual
CAD	Computer-Aided Design
CFs	Carbon Fibers
CFRP	Carbon Fiber-Reinforced Polymer
CNC	Computer Numerical Control
CTE	Coefficient of Thermal Expansion
EF	Environmental Footprint
EoL	End of Life
EU	European Union
FEM	Finite Element Method
FRP	Fiber-Reinforced Polymer
FU	Functional Unit
GRFP	Glass Fiber-Reinforced Polymer
GWP	Global Warming Potential
ISO	International Standard Organization
LCA	Life Cycle Assessment
LCI	Life Cycle Inventory
LCIA	Life Cycle Inventory Analysis
MM	Master Model
PAN	Polyacrylonitrile
PC	Polycarbonate
PU	Polyurethane
RTM	Resin Transfer Molding
R&D	Research and Development
T4L	Tool4Life
USA	United States of America
VOC	Volatile Organic Compound
